# Three-dimensional-printed silk fibroin scaffolds loaded with adipose-derived stem cells prevent post endoscopic submucosal dissection esophageal stricture in a porcine model

**DOI:** 10.1093/rb/rbag057

**Published:** 2026-03-13

**Authors:** Shujun Ye, Daxu Zhang, Xiaonan Shi, Zhanbo Wang, Jingjing Hu, Shuo Zhao, Weilong Li, Jingyi Wang, Weiping Guan, Lianjun Ma, Li Yan

**Affiliations:** The Second Medical Center and National Clinical Research Center of Geriatric Diseases, Chinese PLA General Hospital, Beijing 100853, China; Department of General Surgery, The First Medical Center, Chinese PLA General Hospital, Beijing 100853, China; The Second Medical Center and National Clinical Research Center of Geriatric Diseases, Chinese PLA General Hospital, Beijing 100853, China; Department of Pathology, Chinese PLA General Hospital, Beijing 100853, China; The Second Medical Center and National Clinical Research Center of Geriatric Diseases, Chinese PLA General Hospital, Beijing 100853, China; The Second Medical Center and National Clinical Research Center of Geriatric Diseases, Chinese PLA General Hospital, Beijing 100853, China; The Second Medical Center and National Clinical Research Center of Geriatric Diseases, Chinese PLA General Hospital, Beijing 100853, China; The Second Medical Center and National Clinical Research Center of Geriatric Diseases, Chinese PLA General Hospital, Beijing 100853, China; The Second Medical Center and National Clinical Research Center of Geriatric Diseases, Chinese PLA General Hospital, Beijing 100853, China; Endoscopy Center, China-Japan Union Hospital of Jilin University, Changchun, Jilin Province 130033, China; The Second Medical Center and National Clinical Research Center of Geriatric Diseases, Chinese PLA General Hospital, Beijing 100853, China

**Keywords:** tissue engineering, esophageal stricture, endoscopic submucosal dissection, adipose-derived stem cells, 3D printing

## Abstract

Endoscopic submucosal dissection (ESD) is the preferred treatment for early esophageal cancer. However, extensive mucosal dissection frequently results in esophageal stricture. This study aimed to evaluate the efficacy of Three-dimensional (3D)-printed silk fibroin scaffolds (SFS) loaded with adipose-derived stem cells (ADSCs) in preventing post-ESD esophageal stricture, and to explore the underlying mechanisms. A near-circumferential ESD model was established in pigs, three groups were set: Control, SFS and ADSCs-SFS. The dynamic change of postoperative esophageal healing and stricture formation were monitored endoscopically. On postoperative Day 28, esophageal specimens were collected to measure mucosal contraction rate, followed by histological evaluation of inflammation and fibrosis, as well as mRNA transcriptome sequencing to analyze gene expression and the change of the enriched signaling pathways. On Day 28, the ADSCs-SFS group showed significantly less weight loss and a markedly reduced mucosal contraction rate. Histological examination revealed more complete mucosal regeneration and significantly reduced collagen deposition in the ADSCs-SFS group. Molecular analyses indicated significant downregulation of inflammatory and fibrotic markers in the ADSCs-SFS group. Transcriptome analysis suggested that ADSCs-loaded SFS effectively alleviates esophageal stricture following ESD in pigs, likely through suppression of the PI3K/AKT signaling pathway and reduction of inflammation and fibrosis.

## Graphical abstract

**Graphical Abstract:**
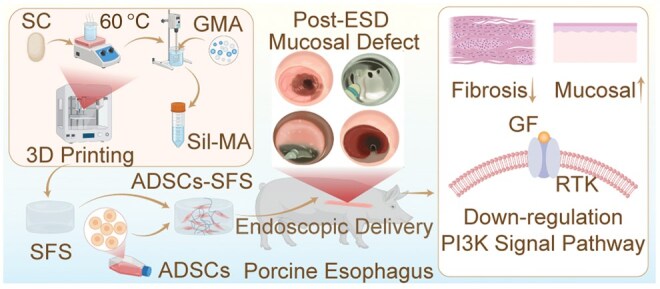

## Introduction

Esophageal cancer ranks as the seventh most commonly diagnosed cancer and the sixth leading cause of cancer-related deaths worldwide [1]. Due to the insidious nature of early-stage symptoms, the majority of patients are diagnosed at an advanced stage, resulting in a poor prognosis [2, 3]. In recent years, advancements in endoscopic techniques have significantly improved the diagnosis and treatment of early esophageal cancer. Endoscopic submucosal dissection (ESD) has become the preferred treatment for early esophageal cancers and precancerous lesions. ESD allows en bloc resection of lesions confined to the mucosa or superficial submucosa under direct endoscopic visualization, minimizing trauma and preserving organ function. Reported en bloc and complete resection rates exceed 90%, demonstrating excellent efficacy and safety [4–7]. However, when the mucosal dissection involves more than three-quarters of the esophageal circumference, the incidence of postoperative cicatricial stricture reaches as high as 90% [8]. Stricture can lead to symptoms such as dysphagia, nausea and vomiting, and may cause complications like reflux and aspiration, severely affecting patients’ quality of life and posing life-threatening risks if left untreated [9]. Once stricture develops, patients often require repeated endoscopic dilatations to maintain esophageal patency, resulting in significant psychological and economic burdens [10]. Therefore, effective prevention of post-ESD esophageal stricture remains a critical clinical challenge.

Current clinical strategies to prevent post-ESD strictures primarily involve mechanical, pharmacological and tissue engineering-based approaches. Mechanical interventions, such as endoscopic balloon dilation (EBD) and esophageal stent placement, offer immediate luminal support but are plagued by high recurrence, stent migration and restenosis driven by granulation tissue overgrowth [11, 12]. Pharmacological prophylaxis, notably topical or systemic corticosteroids administration can reduce stricture formation to some extent, but frequent steroid use is associated with serious side effects including immunosuppression, diabetes and infections [13–15]. Tissue engineering strategies, such as a variety of tissue decellularization strategies including decellularized esophagus [16, 17], decellularized small intestine [18], acellular dermal grafts [19, 20] and other tissues or organs show some efficacy. In parallel, PGA sheets fixed with fibrin glue and the transplantation of autologous oral mucosal epithelial cell sheets, have shown promise in accelerating re-epithelialization [21, 22]. In general, all these approaches continue to face several obstacles and post-operative issues including anastomotic leakage, poor re-epithelization, lack of muscle regeneration, infection and immunogenic responses, strictures in long-term implantation [20]. Thus, there is an urgent need to explore safer and more effective strategies for preventing stricture.

Advances in tissue engineering and regenerative medicine have opened new avenues for preventing post-ESD esophageal stricture. Among these, stem cell-based therapies have garnered significant attention. Adipose-derived stem cells (ADSCs) are considered an ideal cell source due to their accessibility, abundance and ease of isolation [23, 24]. ADSCs secrete various cytokines and growth factors that function through paracrine mechanisms [25], and possess multi-differentiation potential and remarkable capabilities in promoting tissue regeneration [26]. Studies have shown that ADSCs can facilitate angiogenesis, suppress excessive inflammation and reduce scar formation, thereby accelerating the repair of various tissue injuries [27, 28].

However, stem cell therapies face limitations: injected cells often fail to remain localized at the target site, are easily dispersed through body fluids or undergo cell death, resulting in low retention and utilization rates [29]. Preclinical studies in porcine models have demonstrated that MSC-conditioned medium effectively prevents luminal stricture after ESD, suggesting its potential as a therapeutic strategy for humans [30, 31]. Despite these outstanding results, a major challenge remains in guiding stem cells to differentiate toward specific lineages and maintaining their stability and viability *in vivo*. In response, the ‘scaffold-based stem cell delivery strategy’ has emerged in recent years. This approach involves seeding stem cells onto scaffolds before implantation to enhance cell retention and survival at the injury site [32]. An ideal scaffold should exhibit good biocompatibility, suitable degradation rate, mechanical strength and a microstructure conducive to cell adhesion and growth [33]. Silk fibroin (SF), a natural polymer derived from silk, has attracted considerable interest due to its excellent biocompatibility, biodegradability and favorable mechanical properties [34, 35]. However, the esophageal environment poses constraints on biomaterial performance. Soft hydrogel dressing or injectable hydrogel are convenient for endoscopic delivery, but their stability and shape retention can be challenged by the wet surface and repetitive swallowing [36, 37]. In addition, conventional nonprinted biomaterials often possess unpredictable pore architectures, making it difficult to control pore interconnectivity and mechanical response. These lead to nonuniform cell seeding and hindered nutrition transport [38]. In contrast, Three-dimensional (3D) printing provides exquisite control over macroscopic geometry and micro-architecture, enabling controlled porosity and mechanical tunability while improving reproducibility. These features are important for standardization and translation [38, 39]. Accordingly, we designed a model of a silk fibroin scaffold (SFS) by Computer-Aided Design (CAD) technology tailored to support cell growth and match the mechanical environment of the esophagus. High-precision SFS were fabricated using 4K resolution digital light processing (DLP) 3D printing technology. These ADSCs-loaded SFS (hereinafter referred to as ADSCs-SFS) were then transplanted into near-circumferential ESD wounds in a porcine model to evaluate their efficacy in preventing cicatricial stricture and to investigate the underlying mechanisms.

## Materials and methods

### Study design

A total of nine female pigs weighing approximately 35 kg each (purchased from Beijing Fuhao Experimental Animal Co., Ltd.) were included in this study. The experimental protocol was approved by the Ethics Committee of the Chinese PLA General Hospital, and the animal testing license code is 2019YFA010600. Using a randomized controlled design, the animals were divided into three groups (*n* = 3 per group): Control group, SFS group and ADSCs-SFS group. All pigs underwent near-circumferential ESD (covering approximately 3/4 of the esophageal circumference, approximately 8 cm in length). Postoperatively, the ADSCs-SFS group received endoscopically delivered ADSCs-loaded SFS grafts on the wound; the SFS group received blank SFS grafts; and the Control group received no additional treatment. Venous blood samples were collected on Day 0 (pre-operation) and on postoperative Days 1, 3 and 7. Plasma levels of inflammatory cytokines (IL-1β, IL-6, TNF-α) were measured using enzyme-linked immunosorbent assay (ELISA). Endoscopic examinations were performed on postoperative Days 3, 7, 14 and 21 to assess mucosal healing and luminal patency. All endoscopic and histological evaluations were conducted independently by two investigators in a blinded manner. On postoperative Day 28, all animals were euthanized and esophageal tissue samples were harvested for subsequent analyses (including mRNA sequencing, RT-qPCR, WB and immunohistochemical [IHC]).

### Primary culture and characterization of ADSCs

Subcutaneous adipose tissue was aseptically harvested from the abdomen of one-month-old male Duroc pigs, minced and carefully freed from blood vessels and fascial components. The tissue fragments were digested in 0.2% type I collagenase solution (Gibco) at 37°C for 2 h with magnetic stirring. Digestion was terminated by adding DMEM medium (Gibco) supplemented with 10% fetal bovine serum (FBS, Gibco) and the resulting suspension was filtered. After centrifugation, the supernatant was discarded and the cell pellet was resuspended in DMEM complete medium containing 10% FBS and 1% penicillin–streptomycin. The cells were then seeded into culture flasks and incubated at 37°C under 5% CO_2_. When primary cells reached 80–90% confluence, they were passaged using trypsin. Cells from passages 3–4 were used for subsequent experiments. Surface marker expression was analyzed by flow cytometry to confirm ADSC identity. To verify multilineage differentiation potential, ADSCs were induced to undergo adipogenic, osteogenic and chondrogenic differentiation. Successful differentiation was confirmed using Oil Red O staining for adipocytes, Alizarin Red staining for osteocytes and Alcian Blue staining for chondrocytes.

### Preparation of silk fibroin bioink

Approximately 40 g of silkworm cocoons (Bombyx mori) were degummed by boiling in 0.05 M sodium carbonate solution (Macklin, USA) for 40 min, followed by repeated rinsing with distilled water and drying. About 20 g of dried SF was dissolved in 100 mL of 9.3 M lithium bromide solution (Macklin, USA) at 60°C for 1 h to obtain a transparent solution. Subsequently, 6 mL of glycidyl methacrylate (GMA, 424 mM, YuanYe Bio-Technology) was slowly added, and the reaction proceeded under stirring at 300 rpm for 3 h to achieve GMA grafting onto SF. After the reaction, the solution was filtered through a 100 μm filter to remove impurities, then transferred into a dialysis bag (12–14 kDa molecular weight cut-off) and dialyzed against distilled water for 4 days (with water changed every 12 h) to remove LiBr and unreacted small molecules. The dialyzed solution was freeze-dried for 48 h to obtain porous methacrylated silk fibroin (Sil-MA) solid. The Sil-MA solid was dissolved in an appropriate amount of distilled water to prepare a 30% (w/v) Sil-MA solution. Then, 0.2% (w/v) lithium phenyl-2,4,6-trimethylbenzoylphosphinate (LAP, Innochem) was added and thoroughly mixed to obtain the silk-based bioink for 3D printing.

### Preparation of SFS

The SFS were fabricated using a 4K-resolution DLP 3D printer (Shenzhen Guangyunda Optoelectronics, China; physical resolution: 3840 × 2160, optical accuracy: 5 µm). The prepared SF bioink was placed into the printer’s material tank. A 405 nm light source (power density: 21 mW/cm^2^) projected patterns from below to photopolymerize the corresponding regions of the bioink, with each layer exposed for approximately 0.8 s. The cured layers adhered to a build platform that gradually ascended. After each layer was cured, the platform was raised by one layer thickness and the light source projected the next cross-sectional image. This cycle was repeated to achieve layer-by-layer construction of the scaffold. The printing parameters were set as follows: layer thickness of 40 µm, designed pore size of 100 µm and each model layer thickness being an integer multiple of the cured layer thickness to ensure dimensional accuracy. After printing, the resulting SFS were immersed in 100°C water to remove uncured residues, then dried and stored for subsequent use. To quantitatively assess the 3D printability and structural accuracy, the printing fidelity of SFS was evaluated. The actual pore size of printed scaffolds was measured using ImageJ based on SEM images (*n* = 20 pores, randomly selected). The fidelity was calculated by quantifying the deviation of printed dimensions from the CAD design, using the following formula [40, 41]:


$$\mathrm{Fidelity}(\%)=\left(1-\frac{\left|D_{\mathrm{measured}}-\left.D_{\mathrm{designed}}\right|\right.}{D_{\mathrm{designed}}}\right)\times 100\%.$$

### Fourier transform infrared spectroscopy analysis

Fourier transform infrared spectroscopy (FTIR; Frontier, PerkinElmer, Rodgau, UK) was employed to characterize the graft copolymerization reaction between Sil-MA and unmodified SF. A universal attenuated total reflection (UATR) accessory was mounted on the optical stage of the FTIR system. Lyophilized samples of Sil-MA and unmodified SF (2 mg each) were thoroughly mixed and ground with KBr. The KBr-mixed samples of Sil-MA and SF were then separately placed on the detector of the FTIR spectrometer for measurement.

### Proton nuclear magnetic resonance (^1^H-NMR) assay

To determine the degree of methacrylation, ^1^H-NMR spectroscopy was performed using a Bruker 600 MHz AVANCE III HD spectrometer (9.4 T, Bruker Analytik GmbH, Karlsruhe, Germany). A 2.5 mg sample of Sil-MA was dissolved in 500 µL of deuterated water (D_2_O, Sigma-Aldrich), filtered through a 0.45 µm membrane and subjected to measurement. ^1^H-NMR spectra of both SF and Sil-MA were recorded. The degree of methacrylation was defined as the percentage change in the ε-amino groups of lysine residues in SF after methacrylation. The relative reduction was calculated by integrating the methylene group signal of lysine at *δ* = 2.83 ppm. The DoM% was then determined using the following formula [42]:


$$\mathrm{DoM}\%=1–(\frac{\mathrm{Lysineintegratedsignal}\;\mathrm{of}\;\mathrm{functionalized}\;\mathrm{Sil}–\mathrm{MA}}{\mathrm{Lysineintegratedsignal}\;\mathrm{of}\;\mathrm{SF}}\times 100\%).$$

### Mechanical property testing

The compressive mechanical properties of the SFS were evaluated using a universal material testing machine (MTS C42.503Y, MTS Systems Corp., USA). Cylindrical scaffold samples with a diameter of 1.4 cm and height of 1.6 mm were placed on the test stage. The plates compressed the samples at a rate of 5 mm/min until structural failure occurred. The stress–strain curve was recorded, and the stress (σ) and strain (ε) values at the point of fracture were obtained. The compressive elastic modulus (Ec) was calculated from the slope of the linear region of the curve using the formula:


$$\mathrm{Ec}=\frac{\sigma }{\varepsilon },$$

where σ = instantaneous load (*N*)/initial cross-sectional area (mm^2^) of the scaffold, and ε = compression displacement (mm)/initial height (mm) of the scaffold.

### *In vitro* degradation ratio assay

The 3D-printed SFS were fully immersed in 5 mL PBS solution containing collagenase type II at a final activity of 0.4 U/mL (totaling 2 U). The samples were under static incubation at 37°C without periodic replacement of the enzymatic solution. For the control group, scaffolds were incubated under identical conditions in 5 mL of enzyme-free PBS. Samples were collected at 0, 2, 4, 8, 12 and 24 h, freeze-dried and weighed (denoted as *W*_d_). The initial weight at 0 h was recorded as *W*_d0_, and weights at subsequent time points were denoted as *W*_dn_. The *in vitro* degradation ratio (DR*invitro*) was calculated according to the formula [43]:


$${\mathrm{DR}}_{in\;\mathrm{vitro}}(\%)=\frac{W_{d0}-W_{\mathrm{dn}}}{W_{d0}}\times 100\%.$$

### Water absorption test

The initial dry weight (*W*_1_) of the dried SFS was recorded. The scaffolds were then immersed in ultrapure water at 37°C. At predetermined time points (0, 1, 2, 3, 5, 10, 15, 30 and 60 min), the samples were removed, gently blotted to remove surface moisture and immediately weighed to obtain the wet weight (*W*_2_). Unmodified SFS were tested under the same conditions as a control. The swelling ratio (SR) was calculated using the following formula:


$$\mathrm{SR}(\%)=\frac{W_{2}-W_{1}}{W_{2}}\times 100\%.$$

### Scaffold morphology and microstructure observation

The macroscopic morphology and microstructure of the SFS, as well as the adhesion and growth of ADSCs on the scaffolds, were examined using stereomicroscopy and scanning electron microscopy (SEM). After 3 days of co-culture, the ADSCs-SFS constructs were harvested, rinsed three times with PBS buffer (Gibco) and fixed in 2.5% glutaraldehyde for 4 h. The samples were then dehydrated through a graded ethanol series (50%, 60%, 70%, 80%, 90% and 100%; 10 min per concentration, repeated twice), followed by immersion in isoamyl alcohol for 40 min and subsequent treatment with hexamethyldisilazane (HMDS) for 8–10 min. After critical point drying and sputter coating with gold, the microstructural morphology, pore architecture and cellular adhesion and growth on the pore walls were observed under SEM (S-4800, Hitachi, Japan).

### Live/dead cell viability assay

ADSCs were seeded onto sterilized SFS and cultured at 37°C under 5% CO_2_. Cell viability was assessed on Days 1, 3 and 7. Live/dead fluorescence staining was performed according to the manufacturer’s protocol: cell-seeded scaffolds were incubated in PBS containing 5 µM CMFDA (green fluorescence, labeling live cells) and 4 µM propidium iodide (PI, red fluorescence, labeling dead cells) at 37°C for 15–20 min in the dark. After incubation, the samples were washed three times with PBS and imaged under a confocal laser scanning microscope (Nikon A1, equipped with LSM module). Live cells exhibited green fluorescence, while dead cells showed red fluorescence. Three random fields per sample were captured, and the numbers of green and red fluorescent cells were counted using ImageJ software. Cell survival rate was calculated as follows:


$$\text{Survival}\;\text{rate}(\%)=\frac{\text{number}\;\text{of}\;\text{live}\;\text{cells}}{\text{total}\;\text{number}\;\text{of}\;\text{cells}}\times 100\%.$$

Additionally, Z-stack images were acquired at 200 µm intervals along the vertical axis and reconstructed to visualize the overall distribution and density of cells within the scaffold.

### Cell proliferation assay

Proliferation of ADSCs on SFS was quantitatively evaluated using the Cell Counting Kit-8 (CCK-8) assay. ADSCs were seeded onto SFS at a density of 1 × 10^4^ cells per well in a 96-well plate and cultured under 37°C and 5% CO_2_. On Days 1, 3, 5 and 7 after seeding, the culture medium was replaced with 100 µL of fresh medium containing 10% CCK-8 reagent (Dojindo, Japan), followed by incubation at 37°C for 2 h in the dark. Then, 100 µL of supernatant from each well was transferred to a new 96-well plate, and the absorbance (optical density, OD) was measured at 450 nm using a microplate reader (Beckman, Fullerton, CA, USA). Each experimental group included three replicate wells per time point, and OD values from blank scaffold wells (without cells) were used for background subtraction. The relative proliferation rate was calculated as:


$$\text{Relative}\;\text{proliferation}\;\text{rate}=\frac{\mathrm{experimental}\;\mathrm{OD}}{\mathrm{blank}\;\mathrm{OD}}.$$

Independent samples *t*-test was used to compare proliferation rates among groups, with a significance level set at *P *< 0.05.

### *In vitro* cell migration assay

To determine the effect of SFS on ADSCs migration, we evaluated it using vertical cell migration (Transwell chamber assay) *in vitro*. More experimental procedures are available in Supplementary File S1.

### Preoperative preparation for porcine ESD

Animals were fasted for 24 h and deprived of water for 8 h prior to the procedure. Vital signs, including body temperature, heart rate and respiratory rate, were monitored and recorded before anesthesia induction. Sedation was induced via intramuscular injection of ketamine, followed by endotracheal intubation. General anesthesia was maintained with 1.5–2.0% inhaled isoflurane. Vital signs were continuously monitored throughout the procedure.

### Establishment of porcine ESD model

The ESD procedure was performed by an experienced endoscopist (Lianjun Ma, Chief Physician; >800 ESD procedures performed) under general anesthesia. A single-channel high-definition gastroscope (GIF-Q260J; Olympus, Japan) was introduced into the esophagus. Approximately 30 cm from the incisors, a near-circumferential (approximately 3/4 of the circumference) mucosal area was marked with a Dual-knife (Olympus) using coagulation current, extending distally for approximately 8 cm in length. A mixture containing sodium hyaluronate, methylene blue and normal saline was injected into the submucosa via an endoscopic needle to create a submucosal cushion and elevate the mucosal layer. The mucosal layer was then dissected along the marked line using the Dual-knife until the muscularis propria was exposed. Submucosal injection and dissection were alternated to maintain hemostasis and ensure clear visualization. Prominent submucosal vessels were preemptively clipped to prevent bleeding. After creating a near-circumferential mucosal defect (approximately 8 cm in length, involving ∼3/4 of the circumference), the animals were managed as follows:

Control group: The wound was left untreated.

SFS group: The SFS was endoscopically delivered and placed over the wound.

ADSCs-SFS group: The ADSCs-loaded SFS (1 × 10^6^ ADSCs per scaffold) was covered over the wound and the scaffolds were anchored to the esophageal wound site primarily through their inherent adhesive properties.

### Postoperative care and monitoring

A liquid diet was initiated 24 h after surgery, with a gradual transition to a normal diet. Food intake and vital signs were closely monitored in all animals. To prevent postoperative infection, ceftriaxone sodium was administered intramuscularly at 5 mg/kg once daily for seven consecutive days. The dose was selected according to institutional veterinary protocol. Endoscopic examinations were performed on postoperative Days 3, 7, 14 and 21 to evaluate wound healing and luminal stricture. The degree of dysphagia in each group was recorded using the Mellow–Pinkas dysphagia score: 0 points: no dysphagia; 1 point: ability to swallow some solid foods; 2 points: ability to swallow semisolid foods; 3 points: ability to swallow liquids only; 4 points: complete dysphagia.

### Specimen collection

On postoperative Day 28, all animals were euthanized, and the entire esophagus was immediately harvested. Each esophagus was opened longitudinally along one side, flattened and fixed. The degree of stricture was precisely measured. Mucosal contraction rate was used to quantify esophageal stricture, defined as the percentage reduction in the mucosal diameter at the healed wound site compared to the normal mucosal diameter. The residual luminal diameter at the scar site was measured in the blinding manner by three independent observers and the average value was calculated. A portion of the esophageal tissue was fixed in 4% paraformaldehyde for 24 h, followed by paraffin embedding and sectioning for histological analysis. Another portion of fresh tissue was rapidly frozen in liquid nitrogen and stored for subsequent Western blotting (WB), qPCR and mRNA transcriptome sequencing analyses. Mucosal contraction rate (%) was calculated as follows:


$$\mathrm{Mucosal}\;\mathrm{contraction}\;\mathrm{rate}(\%)=(1-\frac{\mathrm{length}\;\mathrm{of}\;\mathrm{short}\;\mathrm{axis}\;\mathrm{at}\;\mathrm{site}\;\mathrm{of}\;\mathrm{maximal}\;\mathrm{constriction}}{（\mathrm{length}\;\mathrm{of}\;\mathrm{short}\;\mathrm{axis}\;\mathrm{at}\;a\;\mathrm{normal}\;\mathrm{mucosal}\;\mathrm{site}\;\mathrm{on}\;\mathrm{upper}\;\mathrm{side}\;+\;\mathrm{length}\;\mathrm{of}\;\mathrm{short}\;\mathrm{axis}\;\mathrm{at}\;a\;\mathrm{normal}\;\mathrm{mucosal}\;\mathrm{site}\;\mathrm{on}\;a\;\mathrm{lower}\;\mathrm{side}）\times 1/2})\times 100\%.$$

### Enzyme-linked immunosorbent assay

Blood samples (5 mL) were collected from the marginal ear vein of pigs on Day 0 (baseline, pre-operation) and on postoperative Days 1, 3 and 7. The blood was placed in EDTA-anticoagulant tubes, gently mixed and centrifuged at 3000×*g* for 15 min at 4°C to obtain plasma supernatant. The plasma levels of IL-1β, IL-6 and TNF-α were quantitatively measured using ELISA, strictly following the instructions of the commercial ELISA kits (Chenglin Biotechnology, Beijing, China).

### Hematoxylin and Eosin and Masson’s trichrome staining

Fixed esophageal tissues were embedded in paraffin and sectioned into 4 µm-thick slices using a Leica SM2000R microtome. Hematoxylin and Eosin (H&E) staining was performed to evaluate tissue morphology and the degree of inflammation. The extent of inflammatory cell infiltration was graded according to previously established criteria [44]: Grade 1, mild focal infiltration; Grade 2, dense focal infiltration; Grade 3, extensive diffuse infiltration involving >50% of the ulcer base. Masson’s trichrome staining (kit purchased from Solarbio) was conducted to assess collagen fiber deposition, following the manufacturer’s instructions. The thickness of collagen deposition in each group was examined to evaluate the degree of fibrosis.

### Immunohistochemical staining

IHC was performed to examine the local expression of proteins associated with inflammation, angiogenesis and fibrosis in the esophageal tissues. The specific procedure was as follows: After deparaffinization in xylene and rehydration through a graded ethanol series, sections were subjected to antigen retrieval in citrate buffer (pH 6.0) at 95°C for 20 min. Endogenous peroxidase activity was blocked by incubation with 3% hydrogen peroxide at room temperature for 15 min. The sections were then blocked with 5% bovine serum albumin (BSA, Gibco) for 30 min at room temperature. Primary antibodies (e.g. anti-α-SMA, collagen type I, etc.) were applied and incubated overnight at 4°C. The following day, after washing with PBS, HRP-conjugated secondary antibodies (1:500, Servicebio) were applied and incubated for 1 h at room temperature. Color development was performed using a DAB substrate, followed by counterstaining with hematoxylin. The sections were dehydrated through a graded ethanol series, cleared in xylene and mounted with neutral resin. Multiple high-power field images were randomly captured under a microscope (Nikon, Japan). The percentage of positively stained brown area was quantified using ImageJ software.

### RT-qPCR

Total RNA was extracted from esophageal tissues using TRIzol reagent (Invitrogen) according to the manufacturer’s instructions. The purity and concentration of the RNA were measured using a NanoDrop spectrophotometer (A260/A280 ratio between 1.8 and 2.0). cDNA was synthesized using a reverse transcription kit (Takara, RR036A) under the following conditions: 50°C for 15 min and 85°C for 5 s. Quantitative PCR was performed using a QuantStudio 7 Real-Time PCR System (Applied Biosystems) with the following protocol: Pre-denaturation at 95°C for 30 s, followed by 40 cycles of 95°C for 5 s and 60°C for 30 s. A melt curve analysis was conducted after amplification to ensure the absence of nonspecific amplification. Each sample was run in triplicate. GAPDH was used as the internal reference gene, and the relative gene expression was calculated using the 2^−ΔΔ^^*Ct*^ method. The primer sequences can be found in the Supplementary File S2.

### Detection of stem cell migration success

To verify the migration and retention of male porcine ADSCs delivered via SFS at the ESD wound site in female pigs, absolute quantitative real-time qPCR targeting the Y-chromosome *SRY* gene locus was performed using a standard curve method [45]. The standard curve was established using ADSCs derived from male pigs. A series of dilutions were prepared to obtain template DNA with known copy numbers. Real-time PCR amplification using these templates yielded a linear relationship between the Cq (quantification cycle) values and the corresponding copy numbers [46]: *Cq* = *a* + *b* × log_10_(copies). Interpretation followed the criteria: LOD = 10, LOQ = 100 copies/reaction which was defined from the dilution series. Results were reported as: ≥ LOQ, ‘x copies/reaction’ (used for statistical analysis); Between LOD and LOQ, ‘Detected, < LOQ’; < LOD, ‘ND’ (Not Detected). (LOD, limit of detection; LOQ, limit of quantification) [47]. A logarithmic *Y*-axis was used in the figures, with reference lines indicated at 10 and 100. On postoperative Day 7, biopsy tissues were endoscopically collected from the transplantation area. Genomic DNA was extracted and subjected to *SRY* qPCR on a QuantStudio 7 Real-Time PCR System. The obtained Cq values were converted into absolute copy numbers based on the slope and intercept of the standard curve, enabling quantification of stem cell presence at the transplant site.

### Western blotting

Frozen tissue samples stored in liquid nitrogen were ground into homogenates and lysed in RIPA buffer on ice for 30 min, followed by centrifugation to collect the supernatant. Protein concentration was determined using the BCA assay, and loading amounts were normalized across samples. Equal amounts of protein were separated by 10% SDS-PAGE and transferred onto PVDF membranes. The membranes were blocked with 5% skim milk at room temperature for 2 h, then, incubated with primary antibodies (e.g. against TGF-β1, collagen type I, α-SMA, phosphorylated AKT, etc.; diluted 1:1000–1:3000) at 4°C overnight. The following day, the membranes were washed three times with TBST (15 min each), followed by incubation with HRP-conjugated secondary antibodies (Beijing Zhongshan Jinqiao Biotechnology, China) at room temperature for 2 h. After additional washes, protein bands were visualized using ECL chemiluminescent reagent and imaged with a gel documentation system (Bio-Rad). Each experiment was repeated three times independently, and band intensities were analyzed using ImageJ software.

### mRNA transcriptome sequencing analysis

mRNA transcriptome sequencing was performed to analyze mRNA expression changes between the ADSCs-SFS and SFS groups on Day 28 of esophageal repair. Total RNA was extracted from porcine esophageal tissues using TRIzol Reagent (Invitrogen Life Technologies). RNA purity and concentration were assessed using a NanoDrop spectrophotometer (Thermo Scientific), while RNA integrity and quantity were evaluated using an Agilent 2100/4200 system. Eukaryotic mRNA was enriched using oligo(dT) magnetic beads and fragmented via divalent cations. First-strand cDNA was synthesized with random hexamer primers, followed by second-strand synthesis using DNA polymerase I. Double-stranded DNA was end-repaired by adding a poly-A tail to the 3′ end. The cDNA library was constructed through PCR amplification. Sequencing libraries were prepared using the PE150 model (Tiangen Co., Ltd., Beijing, China) on an Illumina NovaSeq 6000 platform. After passing quality control, the libraries were sequenced. Differentially expressed genes (DEGs) were subjected to Gene Ontology (GO) functional annotation and Kyoto Encyclopedia of Genes and Genomes (KEGG) pathway enrichment analysis.

### Statistical analysis

Data are presented as mean ± standard deviation (SD) or median (interquartile range), as appropriate. Normality was assessed using Q-Q plots and the Shapiro–Wilk test. For normally distributed data, comparisons between two groups were performed using the independent samples *t*-test, while one-way analysis of variance (ANOVA) with Tukey’s *post hoc* test was applied for multigroup comparisons. For data that were not normally distributed or exhibited heterogeneous variances, the Kruskal–Wallis test coupled with Dunn’s multiple comparisons test was used to determine significance. All statistical analyses were conducted using GraphPad Prism 10.4.1 and R 4.4.2. A *P* values < 0.05 was considered statistically significant, denoted in figures as follows: * means *P *< 0.05, ** means *P *< 0.01 and *** means *P *< 0.001, ns means not significant.

## Results

### Isolation, culture and characterization of ADSCs

Porcine ADSCs were isolated from subcutaneous adipose tissue by collagenase type I digestion and exhibited spindle-shaped morphology and plastic adherence. Flow cytometric analysis confirmed high expression of CD73, CD90 and CD105 (> 95%) with minimal expression of CD34 and CD45 (<3%), consistent with established immunophenotypic criteria for MSCs (Supplementary File S3 and Figure S1A). Furthermore, the multilineage differentiation potential was verified through successful adipogenic, osteogenic and chondrogenic induction, as evidenced by positive Oil Red O, Alizarin Red and Alcian Blue staining (Supplementary Figure S1B–D). These results demonstrate that the ADSCs isolated in this study exhibit characteristic MSC properties and were suitable for subsequent experiments.

### Preparation of Sil-MA

Sil-MA was synthesized through chemical grafting modification of SF with GMA (Supplementary File S4 and Figure S2A). FTIR spectra of Sil-MA (Supplementary Figure S2B) retained the characteristic SF amide I (∼1640 cm^−1^) and amide II (1530 cm^−1^) peaks while exhibiting new absorption peaks at 1238 cm^−1^ and 951 cm^−1^, corresponding to alcoholic hydroxyl and methacrylate vinyl vibrations, respectively. Enhanced absorption at ∼1640 cm^−1^ indicated the introduction of C=C bonds. These changes confirm the successful grafting of GMA onto SF molecules via epoxide ring opening, introducing photo-crosslinkable C=C bonds into the Sil-MA. ^1^H-NMR analysis (Supplementary Figure S2C) confirmed successful modification of GMA, evidenced by the emergence of methacrylate alkene protons (δ 7.04–6.38 ppm), methyl protons (δ 1.23 ppm) and methylene protons adjacent to nitrogen (N-CH_2_) (δ 4.17 ppm). A significant reduction in the ε-methylene protons of lysine (δ 2.83 ppm) was observed, indicating that grafting primarily occurred at lysine residues (Supplementary File S5). The DoM% was calculated to be 55%. These newly emerged or significantly enhanced signals clearly demonstrate the presence of methacrylate groups on the Sil-MA molecules. Crosslinking was initiated by adding LAP to the Sil-MA solution, triggering radical polymerization of the vinyl groups to form a stable covalent network (Supplementary Figure S2D).

### Characterization of 3D-printed silk fibroin scaffolds

**Figure 1:**
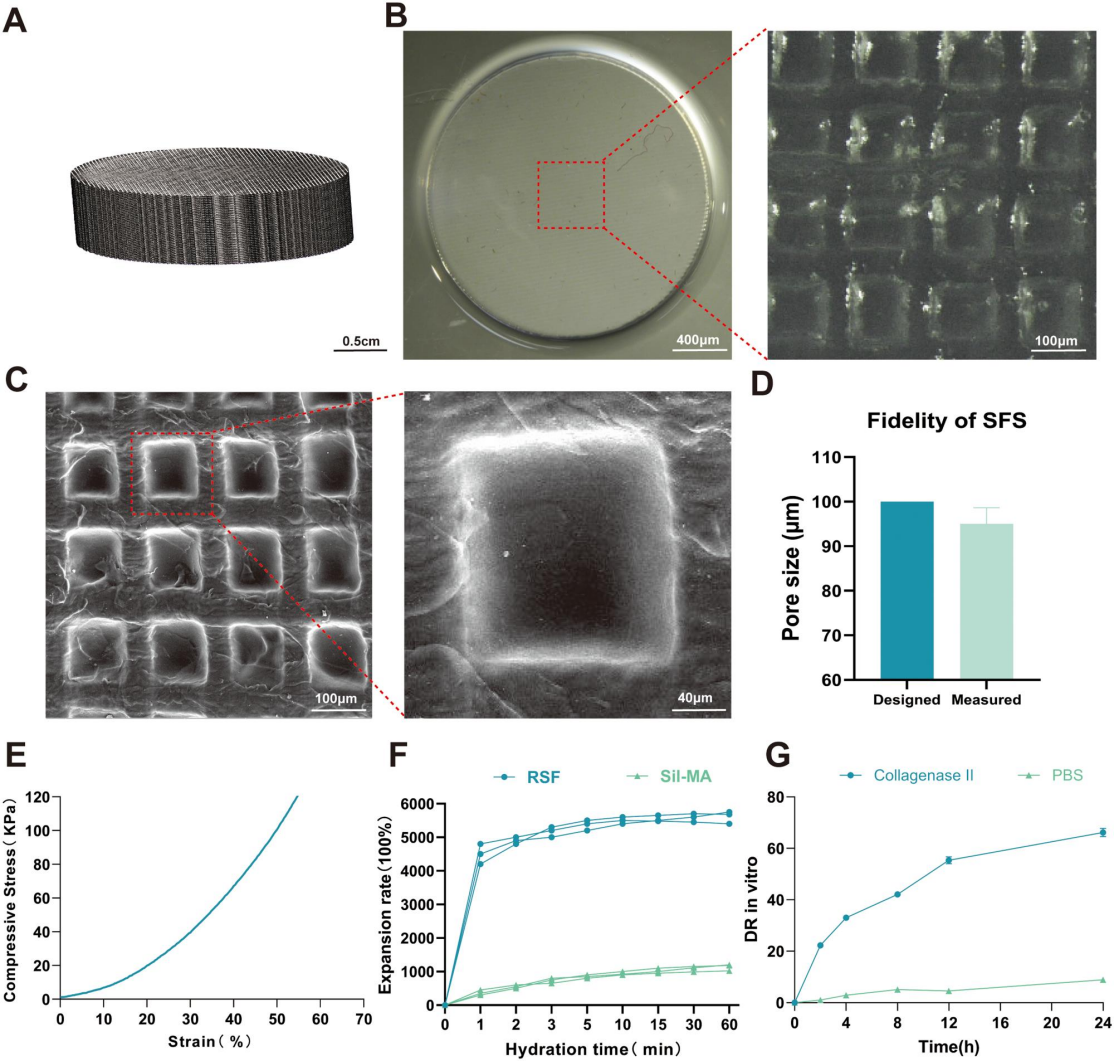
3D printability and physicochemical properties of the SFS. (**A**) The CAD model of SFS; scale bar = 0.5 cm. (**B**) Macroscopic morphology of the SFS. Scale bar = 400 µm, 100 µm. (**C**) SEM image of the SFS; scale bar = 100 µm, 40 µm. (**D**) The fidelity of 3D-printed SFS. Quantitative comparison of the designed pore size (100 µm) and the measured pore size of printed SFS obtained from ImageJ analysis of SEM images (*n* = 20, mean ± SD; CV% = SD/mean × 100%). The designed value is presented as a target reference. (**E**) Mechanical properties of the SFS: the compressive stress–strain curve indicates a compressive elastic modulus of approximately 20 kPa. (**F**) Water absorption and swelling behavior of the SFS. (**G**) *In vitro* degradation rate of the SFS.

A cylindrical scaffold digital model with a diameter of 1.4 cm and height of 1.6 mm was designed using CAD. Based on the average pressure of the esophagus and the requirements for a suitable cell growth environment, the pore size of the model was set to 100 μm (Figure 1A). Under stereomicroscopy, the macroscopic morphology of SFS highly matched the CAD design model, exhibiting a transparent and homogeneous morphology with intact interlayer structures (Figure 1B). SEM further confirmed the existence of a regularly porous architecture with a pore size of 100 μm; 3D reconstruction analysis confirmed an overall scaffold porosity of 45% (Figure 1C). Quantitative analysis indicated that the actual pore size was 95.1 ± 3.5 µm (CV% = 3.7%), closely approximating the designed values. The calculated printing fidelities were 95.1% for pore size. These results demonstrate that the Sil-MA bioink can be printed with high precision (Figure 1D). Compression mechanical testing showed that the SFS exhibited mechanical properties comparable to the native esophageal mechanical environment under axial loading, with a compressive elastic modulus of approximately 20 kPa (Figure 1E). Water absorption and swelling tests revealed that the Sil-MA scaffolds gradually absorbed water and reached a swelling equilibrium within 1–5 min of immersion. In contrast, unmodified regenerated SF rapidly reached its peak water absorption within 2 min and remained stable (Figure 1F). These results indicate that Sil-MA possesses more stable water absorption and swelling properties, enabling it to maintain structural integrity in the moist esophageal environment without excessive expansion or rapid degradation. In the presence of collagenase II, the *in vitro* degradation rate of SFS reached 66% after 24 h, while degradation in PBS was less than 10% over the same period. This demonstrates that the SFS remains stable in a PBS environment but can gradually degrade in an enzyme-rich *in vivo* setting (Figure 1G).

### Biocompatibility of the SFS

**Figure 2:**
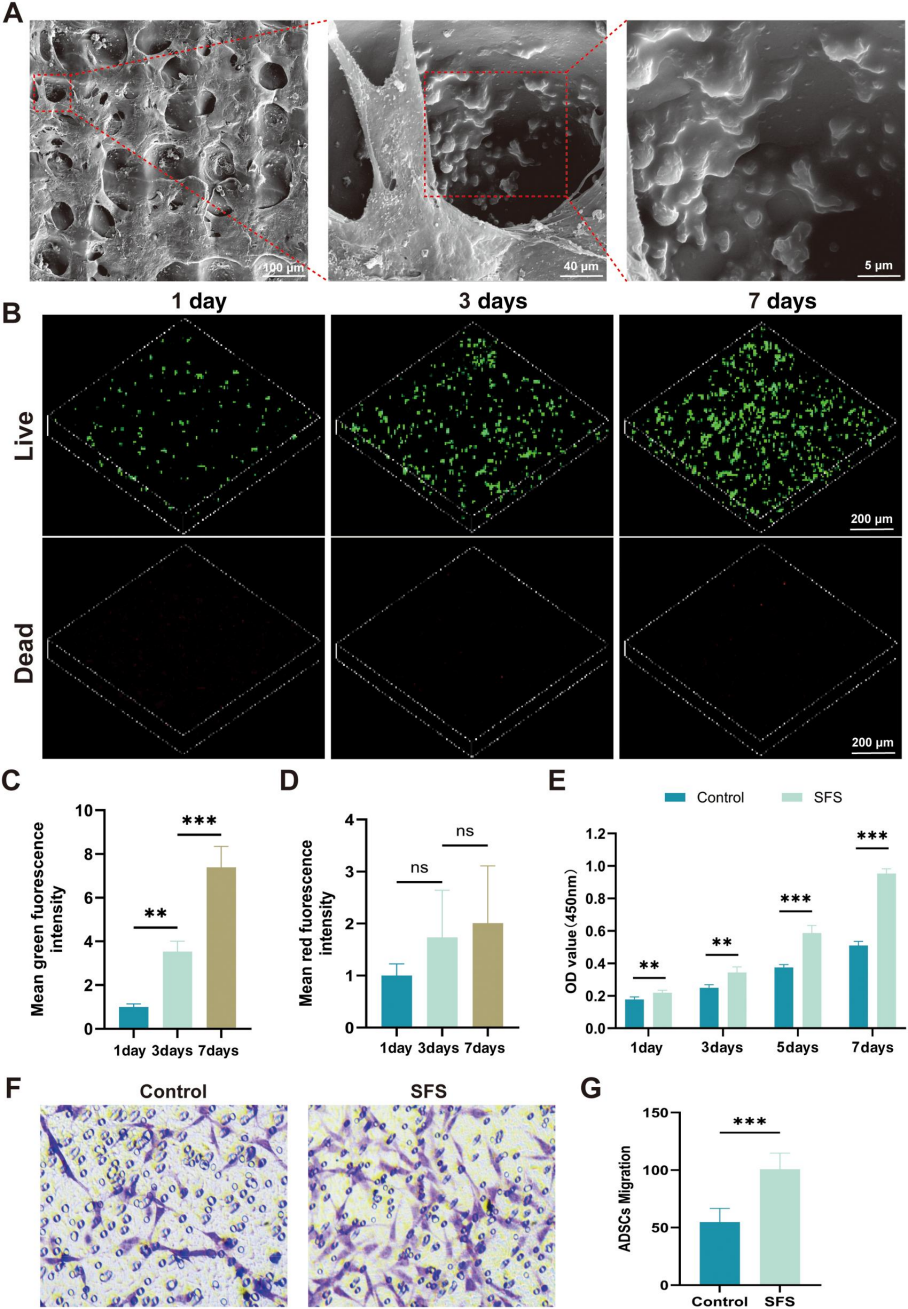
Cytocompatibility and migration-promoting properties of the SFS. (**A**) SEM image showing ADSCs adhering well within the pores of the SFS; scale bar = 100 µm, 40 µm, 5 µm. (**B**) Distribution of ADSCs within the scaffold under live/dead fluorescence staining. Live cells were labeled with CMFDA, dead cells were labeled with PI. Z-stack images were acquired at 200 µm intervals along the vertical (*z*) axis; scale bar = 200 µm. (**C**) Density of live cells over 7 days of culture. (**D**) Density of dead cells over 7 days of culture. (**E**) CCK-8 assay indicating significantly enhanced proliferation of ADSCs on the SFS by Day 7. (**F**) Crystal violet staining of transwell migration assay. (**G**) Quantitative analysis of migrated cell numbers. Statistical analysis: *P < 0.05, **P < 0.01, ***P < 0.001, ns: not significant.

The biocompatibility of the scaffold was evaluated by co-culturing ADSCs with the SFS. SEM observations showed that ADSCs adhered and grew well within the pores of the SFS, with fully extended pseudopodia contacting the pore walls and exhibiting aggregated distribution (Figure 2A). Live/dead fluorescence staining revealed that within 24 h of seeding, the cells had migrated and spread along the scaffold channels, and by Day 7, they were distributed throughout the entire scaffold. The density of live cells on Day 7 was 6 times higher than that on Day 1, and the rate of PI-positive cells remained below 3% throughout the culture period (Figure 2B–D), confirming that the SFS exhibited no significant cytotoxicity. CCK-8 proliferation assays indicated that compared with traditional two-dimensional (2D) culture, ADSCs proliferated significantly more on the 3D-SFS by Day 7 (0.954 ± 0.025 vs. 0.510 ± 0.022, *P *< 0.05, Figure 2E). Transwell migration assay results, including crystal violet staining images and statistical analysis (Figure 2F and G), demonstrated that the number of migrated ADSCs in the SFS group was significantly higher than that in the control group, indicating that the SFS promotes ADSC migration. In summary, the 3D SFS exhibits excellent cytocompatibility, provides a three-dimensional growth environment resembling the extracellular matrix, supports ADSC adhesion and proliferation and facilitates the survival and functional performance of seeded cells.

### The preventive effect of ADSCs-SFS on post-ESD esophageal stricture

**Figure 3:**
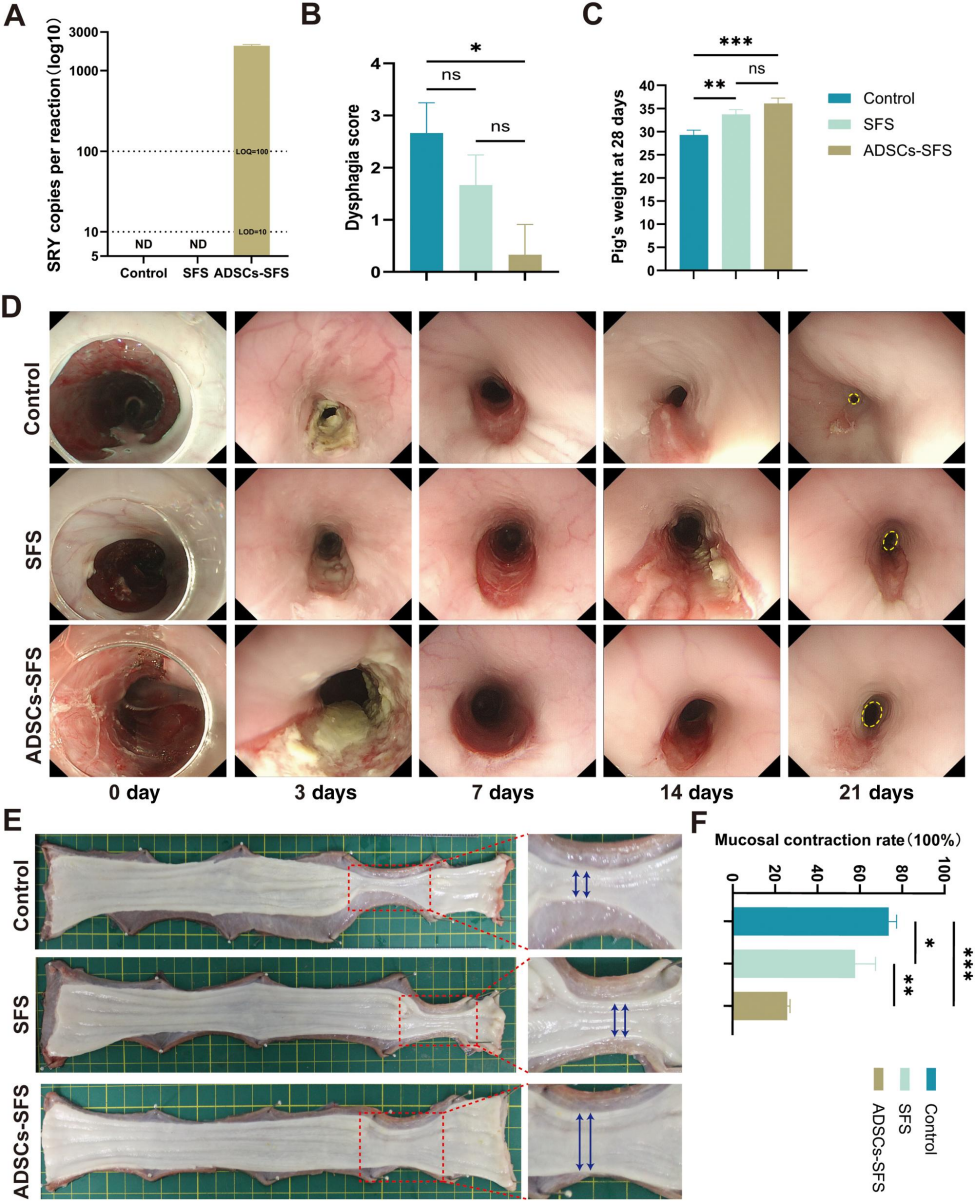
Preventive effect of ADSCs-SFS on post-ESD esophageal stricture. (**A**) Detection of ADSC retention in recipient tissue. (**B**) Dysphagia scores across groups. (**C**) Body weight changes in each group. (**D**) Endoscopic changes in esophageal lumens over time. (**E**) Macroscopic comparison of esophageal specimens. (**F**) Comparison of mucosal contraction rates among groups on Day 28. Statistical analysis: *P < 0.05, **P < 0.01, ***P < 0.001, ns: not significant.

ADSCs isolated from male pigs were seeded onto SFS and transplanted to cover the ESD-induced wounds in female pigs. On postoperative Day 7, wound tissues were endoscopically biopsied for qPCR analysis. Based on the standard curve parameters (In this study, *b* = −3.585, *a* = 39.551, *R*^2^ = 0.999, efficiency > 90%), the ADSCs-SFS group showed significantly higher signals of the male-specific gene *SRY* compared to other groups (Figure 3A), indicating successful retention of the transplanted male ADSCs in the recipient tissues. All animals underwent successful ESD procedures, with wound dimensions covering approximately 3/4 of the esophageal circumference and extending about 8 cm in length. During the 28-day observation period, animals in the ADSCs-SFS group exhibited no significant dysphagia, maintained normal feeding behavior and showed stable body weight. In contrast, the control group gradually developed dysphagia and experienced significant weight loss (*P *< 0.05, Figure 3B and C), while the SFS-only group showed intermediate levels of dysphagia and weight reduction between the other two groups. Endoscopic examinations on postoperative Days 3, 7, 14 and 21 revealed that luminal stricture began to develop by Day 7 in both the control and SFS groups, became pronounced by Day 14, and progressed to severe stricture in the control group by Day 21, with the SFS group showing relatively more moderate but still significant narrowing. In contrast, the ADSCs-SFS group-maintained patent lumens at all time points, with only mild mucosal contraction observed. Some pigs in the ADSCs-SFS group showed white fibrinous exudate and residual scaffold fragments covering the wound at Day 3; by Day 7, no traces of the scaffold remained. To enhance the visual contrast and cross-group comparability, a dashed circular reference ring was overlaid on representative endoscopic images at Day 21 (Figure 3D). Upon harvesting the esophagi on Day 28, mucosal contraction was compared among groups (Figure 3E). The ADSCs-SFS group exhibited a mucosal contraction rate of approximately 25.9%, which was significantly lower than that of the control group (73.7%, *P *< 0.05) and the SFS group (57.9%, *P *< 0.05; Figure 3F). These results demonstrate that ADSCs-SFS significantly promoted ESD wound healing, reduced scar contraction and effectively prevented the development of severe stricture.

### ADSCs-SFS promotes postoperative mucosal regeneration and suppresses inflammatory responses

**Figure 4:**
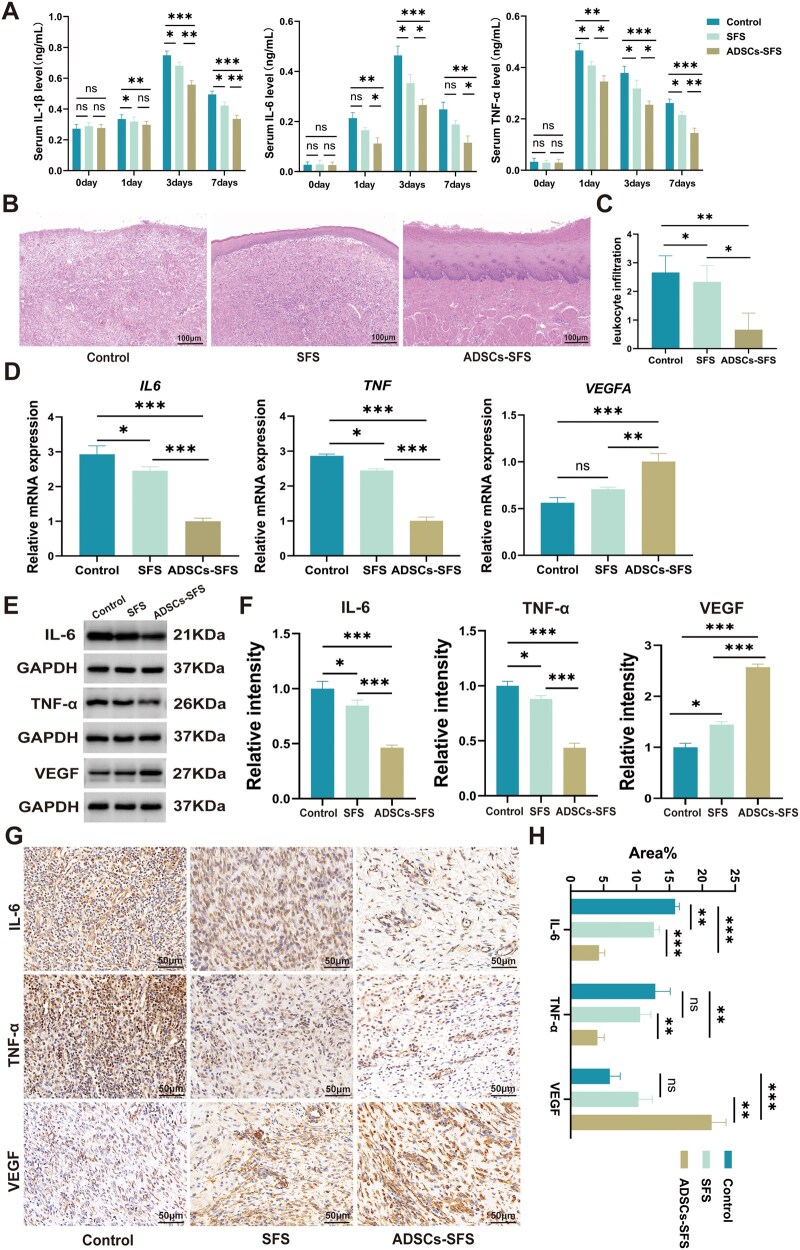
Effect of ADSCs-SFS on postoperative inflammatory response. (**A**) Plasma levels of inflammatory cytokines in the three groups during the acute postoperative phase. (**B**, **C**) H&E staining and inflammation infiltration scores; scale bar = 100 µm. (**D**) Expression of inflammation- and angiogenesis-related genes in wound tissue. (**E**, **F**) Protein levels of inflammatory cytokines and VEGF in wound tissue. (**G**, **H**) IHC results of IL-6, TNF-α and VEGF; scale bar = 50 µm. Statistical analysis: * *P *< 0.05, **P < 0.01, ***P < 0.001, ns: not significant.

Dynamic changes in peripheral blood inflammatory cytokines post-surgery showed that the three groups had similar baseline levels of IL-1β, IL-6 and TNF-α before the procedure. On postoperative Day 1, all groups exhibited a significant increase in cytokine levels; however, the rise was less pronounced in the ADSCs-SFS group compared with the control group. Statistical analysis indicated that ADSCs-SFS significantly suppressed the excessive acute-phase elevation of IL-1β, IL-6 and TNF-α, preventing sustained amplification of the inflammatory response, while the SFS group showed a weaker inhibitory effect (Figure 4A). Pathological analysis of esophageal tissues harvested on Day 28 revealed that H&E staining in the ADSCs-SFS group showed nearly complete regeneration of the mucosal layer, with intact structures of the keratinized, spinous and basal layers, no significant epithelial hyperplasia and only mild inflammatory cell infiltration in the submucosa. The SFS group exhibited thinner newly formed squamous epithelium, with well-developed keratin and spinous layers but incomplete regeneration of the basal layer, accompanied by moderate inflammatory cell infiltration in the submucosa. In contrast, the control group displayed complete loss of the mucosal layer, extensive inflammatory cell infiltration in the submucosa and focal tissue necrosis (Figure 4B). The inflammation score was significantly lower in the ADSCs-SFS group than in the control group (Figure 4C). Compared with the control group, the ADSCs-SFS group showed significantly downregulated gene transcription levels of *IL6* and *TNF* in esophageal tissue (Figure 4D), along with markedly reduced corresponding protein levels (Figure 4E and F). In contrast, the expression of the pro-angiogenic factor VEGF was significantly higher in the ADSCs-SFS group than in the control group, with a modest upregulation also observed in the SFS group (Figure 4D–F). IHC further confirmed that the percentage of positively stained area for IL-6 and TNF-α was significantly smaller in the ADSCs-SFS group than in the control and SFS groups, while VEGF expression was significantly higher in the ADSCs-SFS group compared to the control (Figure 4G and H). These results demonstrate that ADSCs-SFS not only suppressed the acute inflammatory cascade mediated by IL-6 and TNF-α, reducing sustained tissue damage, but also enhanced VEGF levels, promoting neovascularization and providing improved blood supply and nutrients for mucosal regeneration.

### ADSCs-SFS attenuates fibrosis after ESD

**Figure 5:**
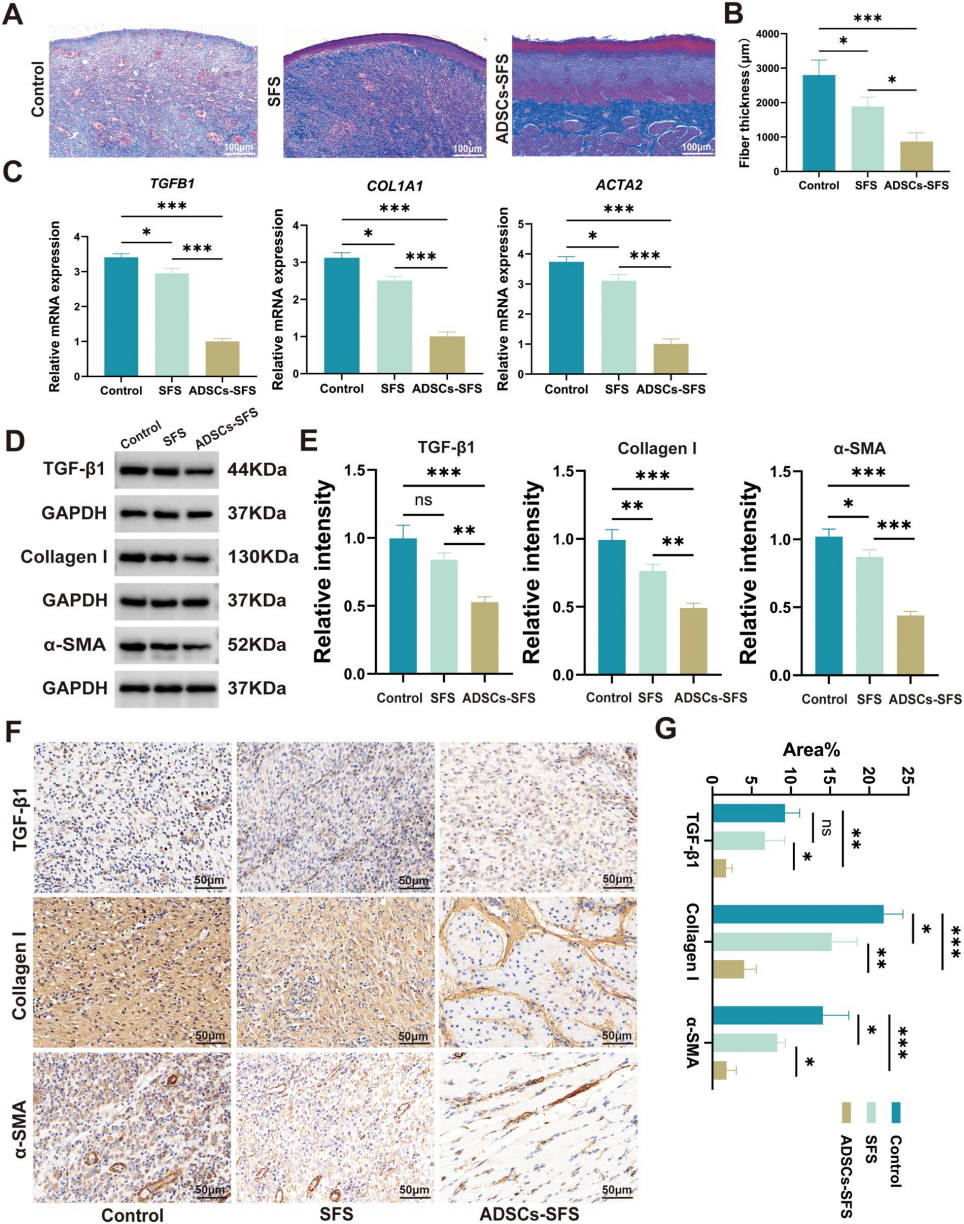
Inhibitory effect of ADSCs-SFS on post-ESD fibrosis. (**A**) Masson’s trichrome staining of wound tissue on Day 28; scale bar = 100 µm. (**B**) Quantitative comparison of collagen fiber thickness. (**C**) Comparison of fibrosis-related gene expression in wound tissues among groups. (**D**) Comparison of fibrosis-related protein expression in wound tissues among groups. (**E**) Grayscale quantitative analysis of WB bands: expression levels of all three proteins were significantly lower in the ADSCs-SFS group than in the control group. (**F**) IHC staining of fibrosis-related proteins in wound tissue; scale bar = 50 µm. (**G**) Quantification of IHC-positive area percentage. Statistical analysis: * *P *< 0.05, **P < 0.01, ***P < 0.001, ns: not significant.

Excessive collagen deposition and fibrosis are major contributors to esophageal stricture following ESD. The degree of fibrosis in each group was evaluated on postoperative Day 28 using Masson’s trichrome staining and collagen quantification (Figure 5A and B). Results showed that the control group exhibited extensive dense collagen fiber deposition in the submucosa of the esophageal wound, with significant fibrotic thickening of the submucosal layer. In contrast, the ADSCs-SFS group showed markedly reduced collagen deposition and much thinner collagen bundles compared with the control group, while the SFS-only group displayed an intermediate level of collagen deposition. RT-qPCR results revealed that the expression levels of fibrosis-related genes (*TGFB1*, *COL1A1* and *ACTA2*) were substantially downregulated in the ADSCs-SFS group compared with the control group, with only mild downregulation observed in the SFS group (Figure 5C). WB analysis similarly demonstrated that protein levels of TGF-β1, collagen type I and α-SMA were significantly lower in the ADSCs-SFS group than in the control group, with the SFS group showing only a modest reduction (Figure 5D and E). IHC further confirmed that the percentage of positive area for TGF-β1, collagen type I and α-SMA was significantly smaller in the ADSCs-SFS group than in the control group (Figure 5F and G). These findings demonstrate that ADSCs-SFS effectively inhibits fibroblast activation and excessive collagen deposition in ESD-induced wounds, thereby attenuating scar fibrosis progression.

### Effect of ADSCs-SFS on signaling pathways during esophageal repair

**Figure 6:**
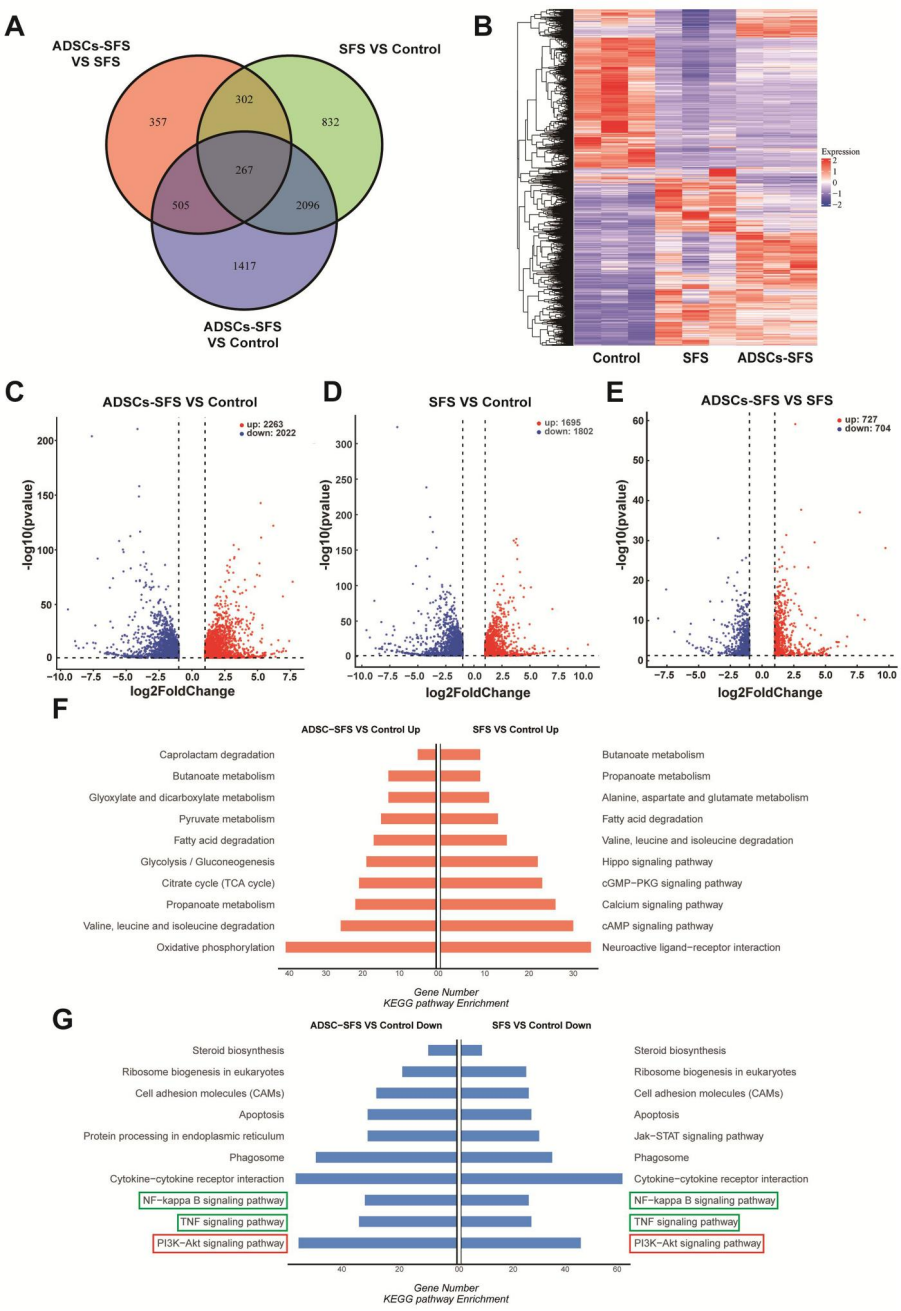
mRNA transcriptome sequencing analysis of the ADSCs-SFS, SFS and control groups. (**A**) Venn diagram showing DEGs among groups. (**B**) Heatmap of DEG expression clustering. (**C–E**) Volcano plots of DEG expression. (**F**, **G**) KEGG pathway enrichment analysis.

To further elucidate the molecular mechanisms underlying the preventive effect of ADSCs-SFS on stricture, mRNA transcriptome sequencing analysis was performed on esophageal tissues from each group on postoperative Day 28. Venn diagram showed that both the ADSCs-SFS and SFS groups had a large number of DEGs, with partial overlap between them (Figure 6A). Cluster heatmap analysis revealed that the gene expression profiles of the ADSCs-SFS and SFS groups were similar but clearly separated from those of the control group (Figure 6B). Compared with the control group, the ADSCs-SFS group exhibited more upregulated and downregulated genes than the SFS group (Figure 6C–E). KEGG pathway enrichment analysis indicated that, compared with the control group, the ADSCs-SFS group showed significant alterations in multiple pathways related to inflammation, immunity, cell proliferation and matrix remodeling; the SFS group also exhibited changes in some of these pathways (Figure 6F and G). Notably, the PI3K/AKT signaling pathway—closely associated with fibrosis—was significantly enriched in both scaffold groups, with an overall downregulation of pathway-related gene expression in the ADSCs-SFS group. The PI3K/AKT pathway is widely recognized as a core driver in fibrogenesis, regulating fundamental processes such as cell proliferation, survival and metabolism. Its sustained activation promotes excessive fibroblast proliferation and survival, as well as aberrant accumulation of extracellular matrix, thereby contributing to the maintenance and progression of fibrotic lesions [48]. These results suggest that ADSCs-SFS may attenuate scar stricture by inhibiting the activity of the PI3K/AKT signaling pathway, thereby modulating the fibrotic process.

### ADSCs-SFS exerts antifibrotic effects by suppressing the PI3K/AKT signaling pathway

**Figure 7:**
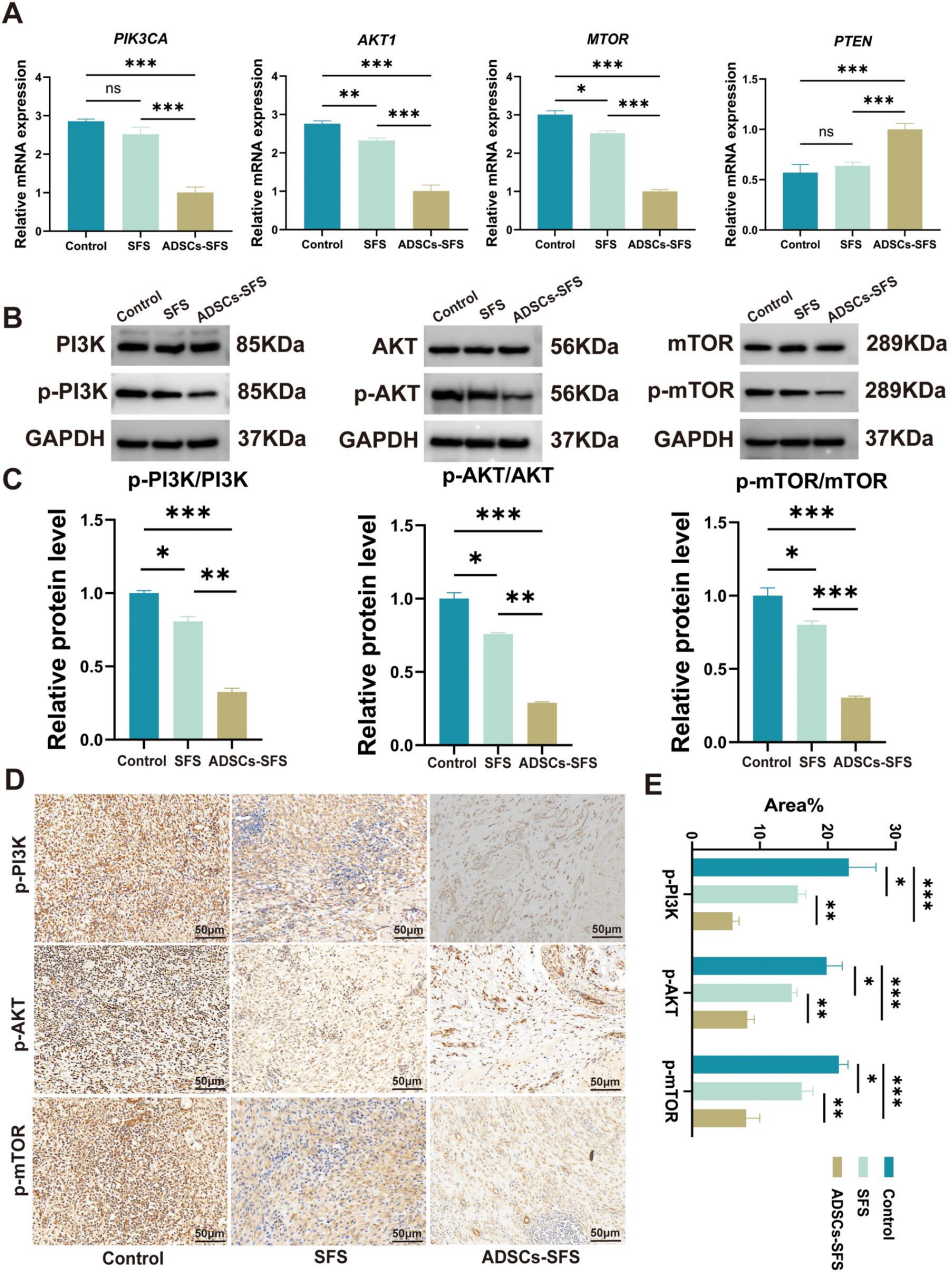
Effects of ADSCs-SFS and SFS on PI3K/AKT pathway activity. (**A**) Expression of *PIK3CA*, *AKT1*, *MTOR* and *PTEN* genes in the control, SFS and ADSCs-SFS groups. (**B**) Protein expression levels of p-PI3K/PI3K, p-AKT/AKT and p-mTOR/mTOR in the control, SFS and ADSCs-SFS groups. (**C**) Quantitative analysis of p-PI3K/PI3K, p-AKT/AKT and p-mTOR/mTOR ratios. (**D**) IHC staining results of p-PI3K, p-AKT and p-mTOR across experimental groups; scale bar = 50 µm. (**E**) Quantification of IHC positive area percentage for p-PI3K, p-AKT and p-mTOR in each experimental group. Statistical analysis: *P < 0.05, **P < 0.01, ***P < 0.001, ns: not significant.

To validate the activity of the PI3K/AKT pathway—a key signaling pathway identified by transcriptome analysis—we performed RT-qPCR, WB and IHC on esophageal tissues from each group. RT-qPCR results showed that, compared with the control group, the ADSCs-SFS group exhibited significant downregulation of mRNA expression of key PI3K/AKT pathway genes (*PIK3CA*, *AKT1* and *MTOR*), while the expression of the pathway inhibitor *PTEN* was upregulated (Figure 7A). WB analysis revealed that protein levels of p-PI3K, p-AKT and p-mTOR were significantly reduced in the ADSCs-SFS group compared with the control group (Figure 7B and C). IHC results further confirmed widespread positive results for p-PI3K, p-AKT and p-mTOR in the control group, whereas the ADSCs-SFS group showed markedly reduced phosphorylation of these proteins (Figure 7D and E). In summary, ADSCs-SFS downregulates the activity of the PI3K/AKT pathway in injured esophageal tissue. Effective interception of PI3K/AKT signaling subsequently restrains the abnormal activation of downstream pro-fibrotic molecules, thereby attenuating fibrosis and preventing stricture formation.

## Discussion

In recent years, several novel approaches have been explored to prevent esophageal stricture. For example, Yano *et al*. [49] utilized biodegradable esophageal stents to prevent post-ESD stricture; however, long-term outcomes were suboptimal, with high stricture recurrence rates and an increased risk of complications such as esophageal-tracheal fistula. Other tissue engineering strategies have included the use of acellular dermal matrix, oral mucosal cell sheets, autologous skin flaps and ADSCs sheet transplantation [22, 50–53]. Nevertheless, even with autologous esophageal mucosal patch repair, the postoperative stricture rate remains as high as 88.9%, accompanied by shortcomings like technical complexity and poor patient compliance [54]. Wei *et al*. [55] reported that covering ESD wounds with a novel tetra-armed polyethylene glycol (tetra-PEG) hydrogel reduced the degree of stricture by approximately 30% compared with the control group. However, challenges remain regarding the long-term efficacy of stricture prevention, as well as the adhesion of the hydrogel to the esophageal wound and sustained drug retention. Therefore, there is an urgent need for more effective and innovative intervention strategies to address post-ESD esophageal stricture.

The targeted delivery of MSCs combined with scaffold materials is considered a promising therapeutic strategy. Scaffolds not only provide mechanical support but also serve as cell carriers, enabling transplanted stem cells to remain at the wound site for extended periods and exert their therapeutic effects. Previous studies have demonstrated that direct injection of ADSCs at the ESD mucosal resection site can reduce mucosal contraction to some extent, though the outcomes are not entirely satisfactory [29]. Based on this, the present study did not include a standalone ADSCs injection group. Our results indicate that ADSCs-SFS significantly reduces the incidence of post-ESD esophageal stricture by mitigating inflammatory responses and fibrosis. The mucosal contraction rate in the ADSCs-SFS group was approximately 48% lower than that in the control group and about 30% lower than that in the empty scaffold group at 28 days postsurgery, demonstrating notable antistricture efficacy. These findings suggest that this strategy holds great promise for preventing esophageal stricture after extensive ESD, particularly for patients who cannot tolerate existing treatments or exhibit poor responses to them.

This study is the first to combine 4K DLP photocurable 3D-printed SFS with ADSCs for the prevention and treatment of post-ESD esophageal stricture. The prepared SFS exhibited precisely controllable structures. We selected Bombyx mori silk, which is easy to get, as the raw material, whose highly ordered β-sheet structure enhances material stability and facilitates purification. After high-temperature degumming, the residual sericin content was less than 0.01% and endotoxin levels met safety thresholds for cellular experiments, ensuring high biosafety [56–58]. The scaffold’s elastic modulus of approximately 20 kPa matched the average pressure environment within the esophageal lumen. GMA was used for the grafting reaction with SF; the methacrylation of SF via epoxide ring-opening reaction increased the degree of methacrylation without producing acidic byproducts [59, 60]. In parallel, the macroscopic geometry and micro-architecture of SFS were selected to balance endoscopic deliverability with *in situ* compatibility in the esophageal lumen. A circular patch with a diameter of 1.4 cm was chosen to allow transoral delivery through commonly used overtube systems (inner diameter 16–17 mm) while minimizing luminal burden [61, 62]. The SFS thickness (1.6 mm) was kept low-profile to minimize luminal burden under continuous peristalsis, maintaining sufficient structural integrity for stable apposition to the mucosal defect without excessive protrusion into the lumen [63]. At the micro-architectural level, a regular square lattice design was adopted to ensure an interconnected pore network and reproducible structural features [64]. The designed pore size (100 µm) was selected to meet permeability for nutrient/metabolite exchange and maintenance of structural stability and local cell residence. It is consistent with prior studies on SFS, pore architecture within this range can modulate MSC behavior and lineage-related responses [38, 65–67]. In this study, high-precision structures were fabricated using DLP printing to ensure faithful reproduction of the designed geometry. The scaffold also exhibited adequate adhesiveness, allowing it to adhere to post-ESD wound sites, thereby facilitating the migration and function of ADSCs at the esophageal injury.

To assess donor-cell persistence, we adopted a sex-mismatched transplantation approach by delivering male-derived ADSCs to female recipients and quantifying donor cells using real-time qPCR targeting the Y-chromosome SRY gene locus in recipient tissue genomic DNA. This strategy has been widely used in regenerative studies because it provides sensitive tracking based on an endogenous male-specific marker [68]. In our study, no overt clinical signs suggestive of graft intolerance (e.g. fever, abnormal vital signs or excessive local inflammation) were observed in any of the experimental animals. The lack of overt rejection is consistent with the generally low immunogenicity and immunomodulatory phenotype for ADSCs, including minimal constitutive expression of MHC class II and key co-stimulatory molecules and the ability to suppress alloreactive immune response [69–71]. In parallel, the ADSCs-SFS group exhibited the lowest levels of IL-1β, IL-6 and TNF-α compared to both the SFS and control groups (Figure 4A). These downward trends confirm that the ADSCs-SFS promotes an anti-inflammatory reaction rather than an exaggerated systemic inflammatory response, validating the immunological safety of this tracking strategy.

In the porcine model of post-ESD stricture used in this study, ADSCs-SFS intervention resulted in the most favorable healing outcomes. The ADSCs-SFS group showed significant improvements in weight recovery, swallowing function and mucosal contraction rate compared to both the SFS and control groups, demonstrating the lowest inflammation levels, the most complete mucosal regeneration and the mildest degree of fibrosis. Transcriptome analysis revealed that ADSCs-SFS significantly suppressed multiple pro-inflammatory and pro-fibrotic signaling pathways, with the most prominent downregulation observed in the PI3K/Akt pathway, along with inhibition of inflammatory pathways such as TNF and NF-κB. The PI3K/Akt signaling pathway plays a key role in promoting fibroblast activation, proliferation and extracellular matrix deposition and is considered a core driver of fibrogenesis. Numerous studies have demonstrated that excessive activation of PI3K signaling can drive abnormal fibroblast proliferation and activation, leading to excessive deposition of extracellular matrix components such as collagen. In pulmonary fibrosis, dysregulation of the PI3K/AKT pathway is closely associated with uncontrolled fibrosis: for example, pro-inflammatory stimuli such as LPS can induce collagen synthesis and fibroblast proliferation through activation of the PI3K/AKT/mTOR pathway, and this process can be reversed using PI3K inhibitors, significantly reducing collagen production [72]. In liver fibrosis following injury, the PI3K/AKT/mTOR pathway is markedly activated in damaged tissues, with levels of phosphorylated PI3K, AKT and mTOR significantly elevated in fibrotic mouse models compared to normal controls [73]. In Crohn’s disease-associated intestinal fibrosis, hyperactivation of PI3K p110δ isoform leads to intestinal smooth muscle thickening, fibroblast hyperplasia and collagen deposition. Genetic knockout or specific inhibition of PI3Kδ significantly reduces intestinal fibrosis and alleviates local chronic inflammation [74]. In our study, both gene and protein expression of classic pro-fibrotic molecules (TGF-β1, α-SMA and type I collagen) were significantly downregulated in the ADSCs-SFS group, consistent with the inhibition of PI3K/Akt signaling, suggesting effective interruption of the fibrotic cascade. It is noteworthy that TGF-β, a central mediator of fibrosis, can activate PI3K/Akt signaling through noncanonical pathways in addition to the classic Smad pathway [75]. Therefore, the significant reduction of TGF-β1 in the ADSCs-SFS group not only directly reduced downstream fibroblast activation but may also have inhibited the fibrotic process at multiple levels by attenuating PI3K/AKT pathway activation. Previous studies have shown that upregulating PTEN, a negative regulator of the PI3K/Akt pathway, can inhibit fibroblast activity in esophageal ESD wounds, reduce α-SMA and collagen production and thereby alleviate stricture formation [76], which is consistent with our findings.

Beyond its antifibrotic effects, the regulation of inflammatory responses by ADSCs-SFS is also a critical factor contributing to improved healing. As shown in Figure 4, during the acute inflammatory phase on postoperative Days 1, 3 and 7, plasma levels of IL-1β, IL-6 and TNF-α in the ADSCs-SFS group were significantly lower than those in the control group, indicating effective suppression of the early inflammatory response. Numerous studies have confirmed that MSCs can secrete various anti-inflammatory mediators and interact with immune cells to attenuate inflammatory signaling pathways [77]. MSCs inhibit the activation of pro-inflammatory transcription factors (NF-κB), reduce the production of pro-inflammatory cytokines including TNF-α and IL-1β in macrophages and tissues and simultaneously enhance the release of anti-inflammatory factors [78]. Transcriptome results from this study also demonstrated inhibition of the TNF and NF-κB signaling pathways in the ADSCs-SFS group. Chronic inflammation is often positively correlated with fibrosis, as recurrent inflammatory damage can induce sustained tissue repair and fibroblast recruitment [79–81]. However, controlling inflammation alone is insufficient to completely prevent fibrosis. For example, in Crohn’s disease, even though anti-TNF-α biologics effectively control inflammation, early studies did not observe a significant reduction in the incidence of intestinal strictures [82]. This suggests that fibrosis is a complex process involving multiple pathways. Effective prevention of scar stricture requires simultaneous intervention in both inflammatory and fibrotic signaling pathways. The ADSCs-SFS strategy mitigates the TNF-α/NF-κB-mediated inflammatory cascade through the immunomodulatory effects of stem cells, reducing persistent inflammatory stimulation of tissues. At the same time, it inhibits pro-fibrotic pathways such as PI3K/AKT, limiting the transformation of fibroblasts into myofibroblasts and excessive deposition of extracellular matrix.

Furthermore, the ADSCs-SFS group showed significantly upregulated VEGF expression and the lowest levels of angiogenesis-inhibiting factors such as IL-6. This combination of ‘reduced inflammation + promoted angiogenesis’ creates an ideal microenvironment for wound healing. Increased VEGF implies enhanced neovascularization, which improves local blood supply and oxygenation, supporting mucosal regeneration and tissue remodeling. Adequate blood perfusion not only accelerates epithelial repair and coverage but may also alleviate hypoxia, thereby preventing excessive activation of hypoxia-induced pro-fibrotic pathways [51]. In summary, the SFS provides favorable physical support for cell migration and proliferation, while the loaded ADSCs dually modulate the healing process through the secretion of various growth factors and anti-inflammatory mediators. ADSCs-SFS concurrently regulates multiple signaling pathways, including PI3K/Akt, TNF and NF-κB, leading to a significant reduction in scar stricture. In contrast, the SFS group, lacking stem cell-mediated bio-regulation, exhibited only mild anti-inflammatory and antifibrotic effects, while the control group, receiving no intervention, developed the most severe persistent inflammation and scar stricture.

Although this study yielded clear experimental results, several limitations should be acknowledged. First, evidence for the inhibition of TNF and NF-κB signaling pathways by ADSCs-SFS primarily originated from transcriptome data, lacking validation at the genetic and protein levels. Future studies could incorporate pathway inhibitors/activators or genetic knockout approaches to further elucidate the causal roles of inflammatory and fibrotic signaling, including the PI3K/Akt pathway, in the antistenotic effects. Second, the sample size in this study was limited, and only 28-day tissues were compared across groups. Subsequent research should expand the sample size and include multiple time points to dynamically observe the tissue repair process and clarify the temporal patterns of inflammatory and fibrotic changes. Finally, this study did not separately evaluate the long-term biocompatibility of SFS in large animal models, which is a limitation. However, SF, as a natural polymer, has been extensively validated for its excellent biocompatibility and biosafety in various animal models and clinical applications [83]. Moreover, no severe inflammatory reactions or systemic adverse events were observed during its application in the porcine esophageal model in this study, which indirectly supports its *in vivo* safety.

## Conclusions

This study demonstrates that endoscopic transplantation of ADSCs-SFS effectively prevents esophageal stricture following near-circumferential ESD in pigs by suppressing inflammation, blocking fibrotic progression and promoting angiogenesis. This strategy shows promise as a novel therapeutic approach for the prevention of post-ESD esophageal stricture, particularly offering hope for patients unsuitable for or intolerant to existing treatments.

## Supplementary Material

rbag057_Supplementary_Data

## Data Availability

Data supporting the results of this study can be obtained from the corresponding author upon reasonable request.

## Management of experimental sites and facilities

Jiang Peng, Institute of Orthopedics, Beijing Key Lab of Regenerative Medicine in Orthopedics, Key Laboratory of Musculoskeletal Trauma & War Injuries PLA, Chinese PLA General Hospital, Beijing 100853, China.
