# Supplementary material for: Three-dimensional-printed silk fibroin scaffolds loaded with adipose-derived stem cells prevent post endoscopic submucosal dissection esophageal stricture in a porcine model
Source: Regen Biomater. 2026 Mar 13;13:rbag057. doi: 10.1093/rb/rbag057 (PMC13135360; doi:10.1093/rb/rbag057)
Supplement: rbag057_Supplementary_Data [file rbag057_supplementary_data.zip › Supplementary File 1.docx]

**In vitro cell migration assay**

A vertical cell migration assay (Transwell assay) was used to evaluate the effects of the scaffolds on ADSCs migration in vitro. 24-well Transwell inserts with an 8µm pore polycarbonate membrane (Corning 3422) were used. Briefly, cells (2 × 10^4^ ADSCs) were seeded in the upper chamber of the Transwell system in serum-free medium. The lower chamber was filled with 5% FBS as the control group, and the 5% FBS + 3D-printed silk fibroin scaffolds was used as the experimental group. Each group was replicated in 3 wells. After 24h of incubation, the chamber was removed and fixed with 4% paraformaldehyde for 30min, and the non-migrated cells were carefully removed from the upper chamber. After washing with PBS, the Transwell membranes were removed, placed on slides, stained with 0.1% crystal violet solution for 15min, washed three times with distilled water, and covered with water-based sealant. Images of migrated ADSCs were observed by light microcopy, and migrated cells were counted in three random microscopic fields per well. The number of migrating ADSCs was calculated using ImageJ software.
