# Supplementary material for: Three-dimensional-printed silk fibroin scaffolds loaded with adipose-derived stem cells prevent post endoscopic submucosal dissection esophageal stricture in a porcine model
Source: Regen Biomater. 2026 Mar 13;13:rbag057. doi: 10.1093/rb/rbag057 (PMC13135360; doi:10.1093/rb/rbag057)
Supplement: rbag057_Supplementary_Data [file rbag057_supplementary_data.zip › Supplementary File 2.docx]

**The primer sequences used in the RT-qPCR experiment of this study**

| **Gene name** | **Primer sequences** |
| --- | --- |
| *SRY* | F：TTCTGCAGTGGGACAGGAAC  R：GGGATATCAACAGGCTGCCA |
| *IL6* | F：GCTGCTTCTGGTGATGGCTA  R：TGAGGTGGCATCACCTTTGG |
| *TNF* | F：GCCCTTCCACCAACGTTTTC  R：CAAGGGCTCTTGATGGCAGA |
| *VEGFA* | F：GTGCCCACTGAGGAGTTCAA  R：AGGCCCACAGGGATTTTCTT |
| *TGFB1* | F：TTACAACAGTACCCGCGACC  R：CCGCTTTCCAGCATTAGCAC |
| *COL1A1* | F：AGCCCTGGTGAAAATGGAGC  R：CACCCTTAGCACCAACAGCA |
| *ACTA2* | F：CTGGACGTACGACTGGCATT  R：GTGGTCACGAAGGAGTAGCC |
| *PIK3CA* | F：CCCAGGTGGAATGAATGGCT  R：GAAAGGCAAAGTCGAGCAGC |
| *AKT1* | F：GTTTCTTCGCCAGCATCGTG  R：CTCCATGCTGTCGTCTTGGT |
| *MTOR* | F：AGCCTTCGTCTATGACCCCT  R：CGGCAGAGTAGGAATCCGTC |
| *PTEN* | F：AGGACCAGAGGAAACCTCAGA  R：AGTATCGGTTGGCCTTGTCT |
| *GAPDH* | F：TCGGAGTGAACGGATTTGGC  R：TGACAAGCTTCCCGTTCTCC |
