# Supplementary material for: Three-dimensional-printed silk fibroin scaffolds loaded with adipose-derived stem cells prevent post endoscopic submucosal dissection esophageal stricture in a porcine model
Source: Regen Biomater. 2026 Mar 13;13:rbag057. doi: 10.1093/rb/rbag057 (PMC13135360; doi:10.1093/rb/rbag057)
Supplement: rbag057_Supplementary_Data [file rbag057_supplementary_data.zip › Supplementary File 3.docx]

**Figure S1.** Isolation and characterization of porcine ADSCs. (A) Flow cytometry results showing high expression of MSC markers CD73, CD90, and CD105, and minimal expression of CD34 and CD45 in the isolated ADSCs. (B-D) Trilineage differentiation potential of ADSCs. Lipid droplets were detected by Oil Red O staining (B, red), mineralized calcium deposits by Alizarin Red staining (C, orange), and cartilage matrix by Alcian Blue staining (D, blue).
