# Supplementary material for: Three-dimensional-printed silk fibroin scaffolds loaded with adipose-derived stem cells prevent post endoscopic submucosal dissection esophageal stricture in a porcine model
Source: Regen Biomater. 2026 Mar 13;13:rbag057. doi: 10.1093/rb/rbag057 (PMC13135360; doi:10.1093/rb/rbag057)
Supplement: rbag057_Supplementary_Data [file rbag057_supplementary_data.zip › Supplementary File 4.docx]

**Figure S2.** Synthesis and characterization of Sil-MA. (A) Schematic diagram of the grafting reaction between SF and GMA. (B) Comparison of FTIR spectra between Sil-MA and unmodified SF. (C) ^1^H-NMR spectra of unsubstituted SF and Sil-MA. (D) Schematic illustration of the photo-crosslinking process of Sil-MA in the presence of the photoinitiator LAP. It also presents the three-dimensional structure of Sil-MA. The green and purple colors respectively represent the alpha helix and beta sheet in the SF secondary structure.
